# Exploring the role of the microbiota member *Bifidobacterium* in modulating immune-linked diseases

**DOI:** 10.1042/ETLS20170058

**Published:** 2017-11-30

**Authors:** Ian O'Neill, Zoe Schofield, Lindsay J. Hall

**Affiliations:** Quadram Institute Bioscience, Norwich Research Park, Norwich, U.K.

**Keywords:** *Bifidobacterium*, immunology, inflammatory bowel disease

## Abstract

The gut-associated microbiota is essential for multiple physiological processes, including immune development. Acquisition of our initial pioneer microbial communities, including the dominant early life genus *Bifidobacterium*, occurs at a critical period of immune maturation and programming. Bifidobacteria are resident microbiota members throughout our lifetime and have been shown to modulate specific immune cells and pathways. Notably, reductions in this genus have been associated with several diseases, including inflammatory bowel disease. In this review, we provide an overview of bifidobacteria profiles throughout life and how different strains of bifidobacteria have been implicated in immune modulation in disease states. The focus will be examining preclinical models and outcomes from clinical trials on immune-linked chronic conditions. Finally, we highlight some of the important unresolved questions in relation to *Bifidobacterium*-mediated immune modulation and implications for future directions, trials, and development of new therapies.

## Introduction

The human gastrointestinal (GI) tract is home to a complex ecosystem of microbes, including bacteria, fungi and viruses, which play a critical role in host health [1,2]. Owing to the ability of these bacteria to interact with the host directly, through physical interactions with the intestinal mucosa, and indirectly, via production of metabolites that can enter the blood stream, there is significant interest in understanding how these bacteria affect our physiology, particularly with respect to immune development and modulation. For many years, there has been a commercial and scientific interest in using beneficial bacteria, such as ‘probiotics’, to positively modulate host health. Probiotics are defined as ‘live microorganisms that, when administered in adequate amounts, confer a health benefit on the host’ [3] and are, for the most part, consisting of strains from the genus *Lactobacillus* and *Bifidobacterium*. Bifidobacteria have been used for many years as supplements to promote host well-being, as their presence, including the high levels observed in infants and stable levels in adults, is associated with a ‘healthy’ state. These bacteria are particularly effective at protecting against infectious diseases [4–7] and modulating immune responses [7,8]. This review discusses *Bifidobacterium* across the life course, and focuses on species and specific strains that have been studied in the context of immune modulation and treatment of disease.

## *Bifidobacterium* across the life course

Bifidobacteria are Gram-positive, heterofermentative, anaerobic bacteria with a distinctive bifid (i.e. Y) shape after which they are named. Originally isolated from the faeces of breast-fed infants by Tissier in 1899, members of the genus *Bifidobacterium* are commonly found in the GI tract of mammals. They have also been isolated from birds, social insects such as honey bees [9,10], and more recently from water kefir [11–13]. There are currently 55 recognised (sub)species of *Bifidobacterium* [14]. Recently, the genomes of representative strains of these taxa have been sequenced allowing greater resolution when classifying potential new strains of bifidobacteria [14–16]. An analysis of 317 core genes, across all 67 representative genomes of Bifidobacteriaceae [including representative strains of the 55 (sub)species of *Bifidobacterium*], classified *Bifidobacterium* into seven phylogenetic clusters: *Bifidobacterium longum*; *Bifidobacterium adolescentis*; *Bifidobacterium pseudolongum*; *Bifidobacterium boum*; *Bifidobacterium asteroides*; *Bifidobacterium pullorum*; and *Bifidobacterium bifidum* [14]. *Bifidobacterium* genomes range from 1.63 Mb (*Bifidobacterium commune* R-52791) to 3.25 Mb (*Bifidobacterium biavatii* DSM 23 969) and have a high G + C content ranging from 65.53% (*Bifidobacterium choerinum* LMG 10 510) to 52.29% (*Bifidobacterium aquikefiri* LMG 28 769). The analysis of the pan genome of *Bifidobacterium* revealed that 38% of all truly unique genes are involved in carbohydrate metabolism, highlighting the importance of this function in the genus [14,16]. Moreover, *Bifidobacterium* possesses a large arsenal of genes encoding glycosyl hydrolases (GHs), with 3989 genes predicted to have this function in the 55 *Bifidobacterium* genomes. The highest number of GH genes was identified in isolates from humans and primates, reflecting the diverse range of dietary carbohydrates consumed by these hosts [14].

In humans, *Bifidobacterium* resides within the GI tract, from birth to old age, which has recently been reviewed by Arboleya et al. [17]. Briefly, bifidobacteria colonise the new-born gut within the first days and weeks after birth, and they represent the most abundant bacterial family ranging from 40 to 80% of the total gut microbiota [18,19]. There is also evidence to suggest that bifidobacteria could begin colonisation of the GI tract *in utero* [20,21]; however, this remains controversial as direct proof for microbial colonisation, and the mechanisms by which bacteria pass from the mother to the foetus remain to be elucidated. Current studies indicate that bifidobacteria are transmitted vertically from the mother's vagina, GI tract, or breast milk. This is supported by findings by Duranti *et al*. [22], who used a novel internal transcribed spacer (ITS) approach trialled previously [23]. Duranti *et al*. found genomically identical bifidobacteria strains in faecal and milk samples from 24 mother-infant pairs. These findings provide initial insights as to why vaginal delivery provides a higher abundance of *Bifidobacterium* in infants, over a caesarean section (C-section) delivery [24,25]. Following birth, breast milk may provide a secondary delivery route for further bifidobacteria [22,26] and additionally drives proliferation of bifidobacteria due to its unique nutritional milieu of human milk oligosaccharides (HMOs), proteins, and lipids [27–29]. Notably, a reduced abundance of *Bifidobacterium* in infants is highly correlated to chronic diseases, including asthma and obesity [30].

As the infant begins to consume solid foods (∼6 months onwards), overall bacterial diversity increases in response to an expanding nutritional environment, and the abundance of bifidobacteria decreases quite rapidly to 30–40% [17,31], and continues to fall gradually during childhood and adolescence. This can be an unstable time period, and *Bifidobacterium* levels can be influenced by puberty, nutrition, and antibiotic use [32–34]. As we reach adulthood, bifidobacterial populations stabilise between 0 and 18%. A further decline is then seen as we enter the elderly phase of life [35], which interestingly also correlates to a decrease in immune function, so-called immunosenescence. Exactly when or why this happens is still unclear, but higher bifidobacteria levels in the elderly are correlated with health and longevity [36,37].

**Figure 1.**
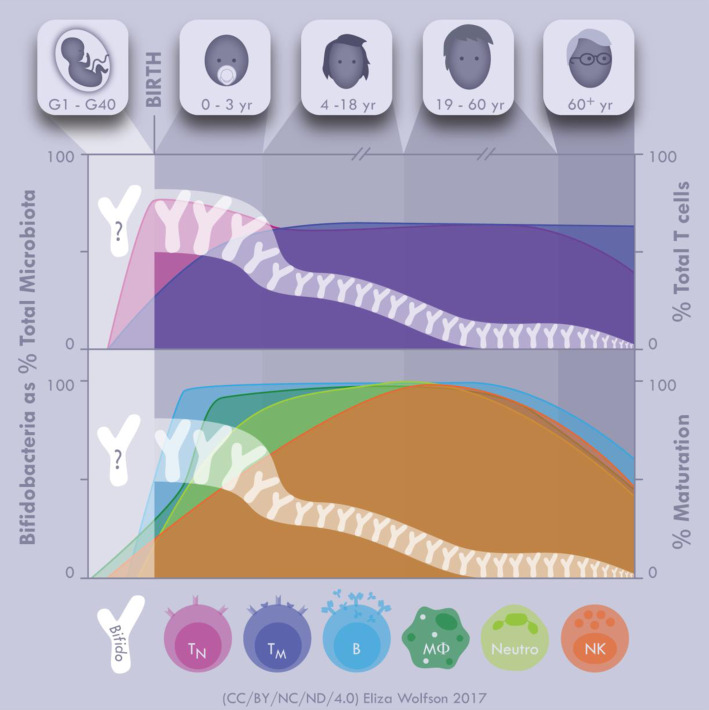
Timeline of bifidobacteria and immune cell maturation throughout life. Although further studies are required to test this hypothesis, this figure illustrates the potential correlation between bifidobacteria and immune cell maturation in early life. The distinct bifid shape (white Y) represents *Bifidobacterium* as a percentage of the total microbiota. DNA analysis indicates that *Bifidobacterium* spp. may cross the placenta, but whether *Bifidobacterium* spp. begins colonisation before birth has not been evidenced, and thus indicated with a question mark. After birth *Bifidobacterium* spp. quickly colonises the infant gut and represents the most abundant bacteria by 2–3 weeks and remains prominent at 40–80% of the total microbiota until solid food is introduced ∼6 months of age. At this age, bifidobacterial populations begin to decrease through childhood and adolescence. It stabilises as we enter adulthood ∼0–18% where it remains for most of our adult life. A further reduction in *Bifidobacterium* levels is then observed as we enter the elderly stages of life. Interestingly, a similar trend is seen with the immune system. Studies have shown that *Bifidobacterium* spp. has an important role in stimulating the immune system. These interactions could potentially occur as early as *in utero* and within the critical early life window after birth linking to the high levels of bifidobacteria also observed at this time period. Illustrated are total number of T cells (top), shown as naive (T_N_) or mature (T_M_), and cell maturation (bottom) for NK cells, B cells, neutrophils (Neutro), and macrophages (Mφ). At birth, a reported 75% of T cells are naive, with 25% mature, indicating potential *in utero* priming. Post-natal immune development is complex, and beyond the scope of this review, however, there is a trend with respect to immune cell maturation; B cells and macrophages are mature by 1 year of age and neutrophils fully mature by 5 years of age. The exception is NK cells that do not mature until 17 years of age, but previous studies have shown that these innate immune cells can be influenced by *Bifidobacterium* spp. Further investigation is required to provide mechanistic correlation, but we hypothesise that bifidobacteria may potentially modulate foetal immune development at the very first stages of life. Figure credit: Eliza Wolfson.

Notably, bifidobacteria levels across the life course align with key stages in immune maturation (Figure 1) and are associated with improved host well-being. However, we are at a relatively early stage in understanding the specific mechanisms whereby *Bifidobacterium* influence this critical homeostatic development and programming, including impact on specific immune populations and signalling pathways. Current studies have focused more on immune-linked diseases, in both patients and preclinical *in vivo* disease models, and thus, this review discusses the role of bifidobacteria in modulating different immune populations and intervention studies in disease cohorts.

## Effects of bifidobacteria on the immune system

**Figure 2.**
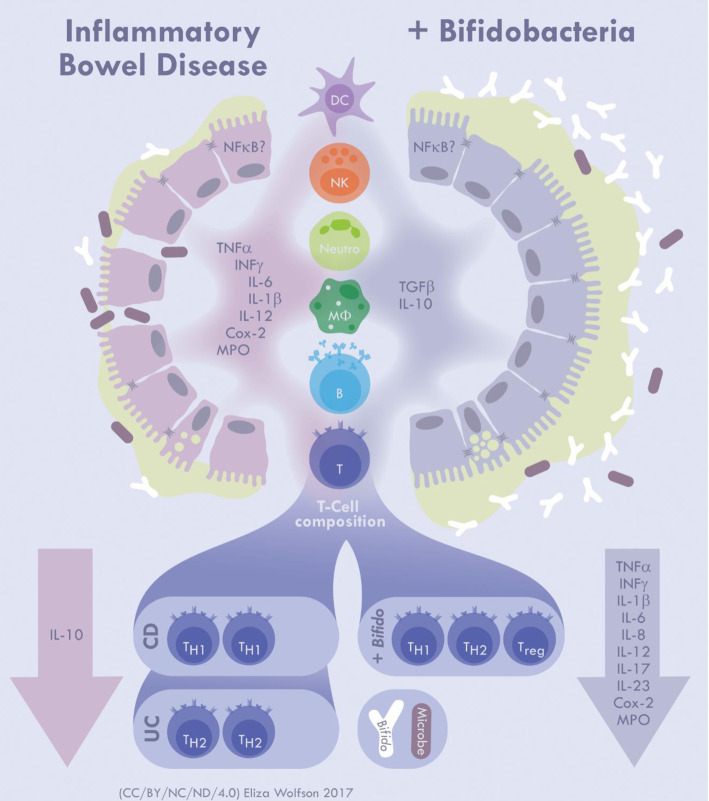
The immune-modulatory effects of *Bifidobacterium* in IBD. IBD is characterised by a damaged or ‘leaky’ IEC barrier and chronic inflammation. A weakened barrier, in tandem with a reduced mucus layer (depicted by light green layer over IECs), enables translocation of luminal microbes into the underlying lamina propria which triggers NF-κB and release of pro-inflammatory cytokines from IECs and immune cells such as macrophages (Mφ) and DCs. Cytokines such as IL-6, IL-23, and TNF-α activate T_H_ cells; CD is marked by an increase in T_H_1 cells, whereas UC is characterised by an increase in T_H_2 cells. In both diseases, there is a reduction in Treg cells, linked to increased IL-12 secretion. *Bifidobacterium* has been shown to reduce levels of key IBD-related pro-inflammatory cytokines such TNF-α, IFN-γ, and IL-1β, and increase the production of IBD protective cytokines TGFβ and IL-10 *in vitro* and *in vivo*, and mucus production *in vitro*. Furthermore, *Bifidobacterium* has been shown to induce Treg cells and reduce restore the T_H_1/T_H_2 cell balance in murine models. Figure credit: Eliza Wolfson.

Data from mouse models and clinical trials indicate that bifidobacteria may have beneficial effects for treating and preventing immune-linked diseases, including gut-associated and systemic conditions. However, we still do not fully understand the mechanisms employed by bifidobacteria to exert their immunomodulatory effects [38]. Studies to date indicate that bifidobacteria have a complex role, having both pro- and anti-inflammatory effects, promoting anti-pathogen immune responses, and modulating immunity in the context of auto-immune or immune-mediated diseases. A significant complication in evaluating these responses lies in the fact that many distinct species and strains of bifidobacteria have been tested, and additionally many of these studies include combination testing with other species or phylum. Furthermore, the cell type, species of animal, model used, and human cohort supplemented also affect immune responses generated [39]. Currently, most mechanistic studies have focused on inflammatory bowel disease (IBD) (Figure 2), allergy, and infection models, reporting bifidobacterial-associated modulation of specific immune cells and their outputs. There are also some limited reports highlighting immune receptor–ligand interactions and downstream signalling events and links to specific bifidobacteria molecules [40,41], such as pili and exopolysaccharide (EPS), on immune responses [5,42,43]. However, it is apparent that the bifidobacteria–immune field requires a greater number of investigations detailing key mechanistic targets and pathways in different immune compartments and immune cell types.

### T cells

From an adaptive immune development perspective, the ratio of T-cell subsets, including T helper1 (Th1), Th2, Th17, and T regulatory cells (Tregs), is key for maintaining homeostasis, while also promoting inflammatory responses in response to appropriate external antigenic stimuli [44]. Notably, irregularities in T-cell responses at different life stages are associated with allergic and chronic inflammatory diseases [45]. Exacerbated Th1 or Th17 responses have been linked to auto-immune disease [46], whereas uncontrolled Th2 responses or reduced Treg responses are associated with allergic reactions [47]. A lack of Tregs is often also found in patients with IBD [48]. Notably, several studies have reported that different strains of bifidobacteria can modulate T-cell responses in immune-driven diseases. In a murine model for chronic allergic asthma, *Bifidobacterium breve* M16-V was shown to increase Treg cell responses (defined as CD4^+^FoxP3^+^ cells) and additionally increase the anti-inflammatory cytokine IL-10 in lung tissue [49]. This was also found to have similar effects as budesonide (i.e. glucocorticoid) treatment. In an ovalbumin-induced food allergy mouse model, the same strain of *B. breve* M16-V (in combination with non-digestible oligosaccharides) was shown to normalise aberrant Th2 responses including a decrease in IL-5 and an increase in IFN-γ, which correlated with a reduction in allergic symptoms [50]. In an IBD-like model, *B. breve* NutRes 204 ameliorated dextran sodium sulfate (DSS)-induced colitis. This was linked to increases in Tregs and decreases in Th17 (CD4^+^IL-17^+^) cell subsets in Peyer's patches of DSS-treated mice and concurrent differential expression of Th1 cells, Th2, and Treg-associated cytokines [51]. Zuo et al. reported an increase in mesenteric lymph node (MLN) Tregs (i.e. CD4^+^FoxP3^+^ cells) in healthy Balb/c mice, and a reduction in Th1-associated cytokines, including IFN-γ and TNF-α. An increase in Treg-associated FoxP3 and anti-inflammatory cytokines IL-10 and TGF-β expression in the MLNs during trinitrobenzene sulfonic acid (TNBS) colitis was also observed [52]. The importance of IL-10 in modulating T-cell responses was further demonstrated in an interesting study where a recombinant *B. longum* NCC2705, producing human IL-10 from a plasmid, was shown to ameliorate colitis in mice by increasing Treg cells and decreasing Th17 cells [53]. However, the use of genetically modified *Bifidobacterium* in humans is a significant regulatory issue, and thus, more in-depth preclinical trials are required to identify the efficacy of these strains and inform regulators.

### Dendritic cells

A potential mechanism whereby bifidobacteria induce T cells may be through dendritic cells (DCs), via antigen presentation and stimulation of antigen-specific T cells. Jeon et al. [54] observed that CD103^+^ DCs isolated from the lamina propria (LP), and stimulated with *B. breve* YAKULT strain, and co-cultured with naive splenic CD4^+^ T cells, lead to IL-10 production and expression of *cMaf*, *Ahr*, and *Il21*, markers of type 1 regulatory T cells. Moreover, this effect was abolished in CD103^+^ DCs from Il10^−/−^, Tlr2^−/−^, and Myd88^−/−^ mice. Konieczna et al. [55] determined that *B. longum* subsp. *infantis* 35 624 increased numbers of CD103^+^ retinaldehyde dehydrogenase (RALDH)^+^ DCs within the LP of mice and that this was associated with decreased Th1 and Th17 cells within the LP, and improved colitis outcomes. Furthermore, in a mouse allergic response to bovine β-lactoglubulin model, *B. longum* BBMN68 induced both CD4^+^CD25^+^Fox3^+^ Treg cells in the MLNs and CD103^+^ DCs in Peyer's patches, which restored the Th1/Th2 balance. Additionally, *ex vitro* experiments indicated that DCs from *B. longum* BBMN68 fed mice also increased TGF-β, IL-10, IFN-γ secretion, and reduced IL-4 secretion from CD4^+^ T cells, further highlighting the indirect role, via DCs, that bifidobacteria have on T-cell cytokine secretion [56].

### Epithelial cells

As bifidobacteria reside within the GI tract, intestinal epithelial cells (IECs) represent a key immune cell type for bifidobacteria-associated modulation. IECs are fundamental for maintaining barrier function during homeostatic conditions, and many different species and strains of *Bifidobacterium*, or their metabolic products, have been shown to increase epithelial cell integrity *in vitro* and *in vivo* [57–59]. In the context of disease, IBD patients, who also have reduced bifidobacteria levels [60], display what is called pathological cell shedding. This is characterised by redistribution of tight junction (TJ) proteins, such as Zonula occludens-1 (ZO-1) and E-cadherin, and increased apoptosis of the epithelial cells at the villus tip, resulting in excessive cell shedding into the lumen [61,62]. In a mouse model of epithelial cell shedding, *B. breve* UCC2003 was shown to reduce the number of apoptotic IECs and corresponding apoptosis signalling molecules. This was mediated via the bifidobacterial EPS capsule and host immune-associated adaptor protein MyD88 [40]. In an IBD-like experimental model, administration of *B. longum* subsp. *longum* 7952, but not *B. longum* subsp. *longum* 372, enhanced expression of TJ proteins in the epithelial layer, which was associated with reduced development of DSS-induced symptoms [63]. This was further highlighted by Hsieh et al. [57] who also showed that only some species and strains of *Bifidobacterium* prevented TNF-α-induced disruption of the epithelial barrier, and promoted tight junctions which, *in vitro*, was attributed to TLR2, but is yet to be defined *in vivo*. These studies emphasise the importance of the species and even the strain of *Bifidobacterium* that is used. In a necrotising enterocolitis (NEC, which is also linked to epithelial barrier disruption) mouse model, *B. breve* was shown to up-regulate TJ proteins Claudin 4 and Occludin [51], and a non-specified species of *Bifidobacterium* increased ZO-1 in a rat NEC model [8]. Studies have also shown that the effects of bifidobacteria are only exerted, or are increased, when live bifidobacteria are used. Grimm et al. [64] showed that the beneficial effects of *B. bifidum* S17 in DSS colitis were seen from only live and not UV-killed bacteria, and Hsieh et al. [57] showed that only live *B. bifidum* had a restorative effect on a TJ impaired Caco-2 cell monolayer. They found that acetate and formate were produced more by B. bifidum than by *B. adolescentis.* Whether it is necessary for bacteria to be ‘alive’ to be effective remains a matter of debate, but differences in structure and components could hold key findings for future therapeutic development.

### Other cells types

Currently, there are limited studies examining the role of bifidobacteria with other immune populations. Kawahara et al. [65] reported that supplementation with *B. longum* MM-2 was linked to increases in natural killer (NK) cell activity, potentially via an increase in NK cell-activating cytokines such as IL-18, and correlated with anti-influenza virus responses. In an obesity-associated inflammation model, *B. pseudocatenulatum* CECT 7765 reduced B-cell (CD19^+^) and pro-inflammatory macrophages (F4/80^+^CD11c^−^CD206^+^), as well as increasing Treg responses, which correlated with reduced body weight gain and improved glucose tolerance [66]. Recently, *B. breve* pre-treatment was shown to significantly decrease the total inflammatory cell number, including decreasing the relative number of eosinophils and neutrophils in a murine airway inflammation model [49].

Overall, these studies indicate that bifidobacteria may have beneficial effects on inflammatory and immune-driven diseases via regulation of specific immune cells and cellular networks, including cytokines (details on *Bifidobacterium*-associated cytokine modulation are shown in Table 1). The implications that bifidobacteria are important modulators of immune responses during disease, both locally and systemically, therefore make them attractive therapeutic targets. Bifidobacteria possess many proteinaceous factors, such as EPS and sortase-dependent pili, that modulate immune responses. This includes the presence, on some strains, of a surface-associated EPS, which has been shown in both *B. breve* UCC2003 and *B. longum* subsp. *infantis* 35624 to modulate innate immune cells, such as neutrophils, macrophages, and peripheral monocytes [5,43]. An EPS deletion mutant in *B. breve* UCC2003 induced more pro-inflammatory cytokine secretion from splenocytes and also increased the number of Ly6G^+^ neutrophils, F4/80^+^ macrophages, DX5^+^/CD3^+^ NK cells, and CD19^+^ B cells in the spleen of treated mice compared with mice treated with the wild-type strain [5]. Similarly, an EPS deletion mutant of *B. longum* subsp. i*nfantis* 35624 stimulated more IL-12p70, IL-17, and IFN-γ from peripheral blood mononuclear cells than the wild-type strain. *B. bifidum* PRL2010 expresses sortase-dependent pili, which when heterologously expressed in a *Lactobacillus lactis* strain induced a higher TNF-α and IL-10 response compared with the non-piliated *L. lactis* strain in a U937 macrophage cell line [42]. A similar response was seen in a murine TNBS colitis model when mice were pretreated with *B. bifidum* PRL2010 [79]. Despite these insights, further studies to elucidate these, and other mechanisms used by bifidobacteria to regulate the immune system, are required. This could include expanding studies to cover exploration of other immune-linked conditions (e.g. inflammatory arthritis), important cell types, specific signalling pathways, and bifidobacteria components or metabolites, and is critical for designing new bacteriotherapies or ‘probiotics’ (Box 1). This may offer a more targeted or personalised approach for patients, as there does not appear to be a one-strain-fits-all scenario.

Box 1. **Areas for exploration in *Bifidobacterium*-immune interactions, and potential experimental tools/approaches that could be used to uncover key mechanisms involved**

16S rRNA (metataxonomic profiling), whole genome sequencing (WGS), global RNA sequencing (RNASeq), knockout (KO).

**Key questions**

• Does bifidobacteria modulate immune responses directly or indirectly (i.e. via wider microbiota modulation)?

• What are the specific strains and species that regulate immune modulation?

• What are the specific components and metabolites that mediate beneficial effects?

• Does bifidobacteria modulate diverse immune cell populations?

• What cell-associated receptors and downstream signalling events are involved in pro- and anti-inflammatory events?

• Does *Bifidobacterium* modulate immune development across the life course, from *in utero* to old age?

• How does bifidobacteria modulate dysregulated immune-linked conditions? Is it via similar pathways as observed in homeostasis?

**Experimental approaches/tools**

• Mono-colonised or defined gnotobiotic models

• Novel cell models to study cross-talk

• In-depth genomic (e.g. WGS) and phenotypic characterisation on key strains and combination studies

• Comparative WGS analysis and transcriptional profiling (e.g. RNASeq) of *Bifidobacterium* and utilisation/development of molecular tools to test key mutants

• Profiling immune populations with flow cytometry and transcriptomics and use of cell-specific mouse KO models

• Use of network analysis and systems biology to define the specific pathways involved

• Human cohort studies and use of life stage-specific (e.g. neonatal) *in vivo* models and immune readouts

• Characterise responses in homeostasis (i.e. ‘healthy’) and correlate to clinically relevant disease models and patient/volunteer cohorts

**Table 1:** Effect of *Bifidobacterium* on cytokine secretions *in vitro* and *in vivo*

*Bifidobacterium* species	Cytokine	Cell type	Model	Ref.	Method
*B. longum*	Low levels of IL-12	Splenic cells	Splenic cells from Balb/c cultured with heat-killed microorganisms (1 µg/ml) for 2 days	[67]	ELISA
*B. breve*	Low levels of IL-12p70				
*B. adolescentis*	Low levels of IL-12p70				
*B. longum*	↓TNF-α, ↑IL-10 ↓IFN-γ	PBMC from coeliac patients	PBMC treated with faecal contents from coeliac disease patients	[68]	ELISA
*B. bifidum*	↓TNF-α, ↑IL-10 ↓IFN-γ				
*B. adolescentis* IM38	↑TNF-α, ↑IL-1β, ↑IL-10, ↓IL-17	Caco2 and mouse peritoneal macrophages	High-fat diet-induced obesity	[69]	ELISA
*B. infantis* 35 624	↓TNF-α	PBMC	LPS-stimulated PBMC from chronic fatigue syndrome, UC and psoriasis patients	[70]	ELISA
*B. bifidum*	↑IL-8	T84 and Caco2 cells	LPS-stimulated cells	[71]	ELISA
*B. infantis* 35 624	↓IFN-γ, ↓IL-12, ↓TNF-α	Splenocytes	Mouse IL-10 KO colitis model. Splenocytes stimulated with *S. typhimurium*	[72]	ELISA
	↓IFN-γ, ↓TNF-α	Mononuclear cells from PP			
*B. longum*	↓IL-1α, ↓TNF-α	Mucosal biopsies	UC patients treated with bifidobacteria	[73]	ELISA
*B. infantis* 35 624	↑IL-10, ↑TGF-β	MLN	Isolated from UC and CD patients	[74]	ELISA
	↑IL-10, ↑TNF-α	PBMCs			
	↑IL-10	MLN-DCs			
	↑IL-10, ↑TNF-α	PBMC-DCs			
*B. bifidum* BGN4	↓IFN-γ, ↓TNF-α	Splenocytes	T-cell transfer model	[75]	ELISA
*B. breve* Yakult	↑IL-10	PBMC	PBMC isloated from UC patients	[76]	ELISA
*B. breve* Yakult	↓IL-8	HT-29	TNF-α-stimulated HT-29	[76]	ELISA
*B. bifidum* Yakult					
*B. bifidum* S17	↓IL-1β, ↓IL-6	Colonic cells	TNBS-induced colitis	[77]	ELISA
*B. lactis* Bb12	↑IL-10, ↑TGF-β	PBMC	PBMC isloated from UC patients	[78]	ELISA
*B. breve* (*BM12/11*, *BM13/14*)	↑IFNγ ↑TNFα,	PBMC	PBMC isolated from healthy donors	[39]	Cytokine Bead Array
*B. animalis* subsp. *lactis* (*BB-12*) and	↑IFNγ ↑TNFα,	PBMC	PBMC isolated from healthy donors		
*B. bifidum* (*KCTC5082*)	↑IFNγ ↑TNFα,	PBMC	PBMC isolated from healthy donors		
*B. bifidum* (IF10/10, A8, DSM20239 and LMG13195)	↑IL-17 ↓IFNγ ↓TNFα,		PBMC isolated from healthy donors		

Abbreviations: UC, ulcerative colitis; PBMCs, peripheral blood mononuclear cells; CD, Crohn's disease; LPS, lipopolysaccharide; PP, Peyer's patches; ↑, increased levels; ↓, decreased levels.

## Bifidobacteria supplementation in patients — evidence from clinical trials

Disturbances in the microbiota are linked to an ever-growing number of immune-linked disease states including IBD, atopic allergy, arthritis, and obesity [80]. Therefore, there is a significant interest in treating these diseases through microbial or ‘probiotic’ supplementation of patients, including with *Bifidobacterium*. Many clinical trials use combinations of *Lactobacillus* and *Bifidobacterium*; however, for this review, we will discuss only studies where *Bifidobacterium* (single or multiple species) were administered as the sole bacteria and/or in combination with a prebiotic (Table 2).

**Table 2:** Use of *Bifidobacterium* in clinical trials

Type of study	No. of subjects	Age	Characteristics of subjects	Probiotic strain	Medication?	Intervention time	Colonisation?	Main outcome	Ref.
RDBPCT	18	24–67 years	Patients with active UC	*B. longum* (2 × 10^11^ CFU) plus 6 g Synergy 1	Yes — steroids (10), immunosuppressants (12), 5-ASA (10)	Twice daily for 28 days	qPCR on biopsies	Short-term treatment improved the full clinical appearance of chronic inflammation in patients with active UC. Reduction in mRNA of TNF-α in the Bif treatment group	[73]
RCT	120	36 ± 16 years (mean)	Patients on remission or with mildly active UC without a history of operation for UC	*B. longum* (2 × 10^9^ CFU) plus 4 g psyllium	Yes — aminosalicylates and/or prednisolone	Twice daily for 28 days	No data	Reduction in CRP in synbiotic compared with Bif and prebiotic-only groups. Synbiotic treatment improved the quality of life better than Bif or prebiotic treatment based on patient questionnaires	[81]
RDBPCT	35	18–79 years	Patients with active CD	*B. longum* (2 × 10^11^ CFU) plus 6 g Synergy 1	Yes — steroids (9), 5-ASA (14), azathioprine (6), mercaptopurine (1), elemental (1) PPI (1)	Twice daily for 183 days	qPCR on biopsies	Bif group had reduction in CD activity index and histological scores and reduction in TNF-α	[82]
RCT	41	45.5 (mean)	Patients with mild-to-moderate UC	*B. breve* strain Yakult (1 × 10^9^ CFU) plus 5.5 g GOS	Yes — salazosulfapyridine, 5-ASA, steroids	Once daily for 365 days	Bacterial counts	A significant reduction in endoscopy score after treatment in the synbiotic group. Not difference in the endoscopy score between control and synbiotic treatment	[83]
RDBPCT	22	18–75 years	Patients with mild-to-moderate UC and CAIA ≥3	*B. longum* subsp. *infantis* 35 264 (1 × 10^10^ CFU)	Yes — 5-ASA (22)	Once daily for 6 weeks	No data	Reduction in plasma CFP and IL-6 levels in the Bif group compared with placebo (no significant reduction compared with pre-treatment)	[70]
RDBPCT	56	44 ± 14 years (mean)	Patients with mild-to-moderate UC and CAIA ≥3–9	*B. longum* 536 (2–3 × 10^11^ CFU)	Yes — 5-ASA (53), prednisolone (17), azathioprine (14)	Three times daily for 8 weeks	No data	Reduction in UCDAI score compared with baseline in the Bif treatment group. No significant difference in UCDAI scores between placebo and control following treatment. A significant decrease in EI score in the Bif group when compared with baseline	[84]
RDBPCT	27	1.3–2.0 months	Manifested atopic eczema during exclusive breast-feeding and who had no exposure to any infant or substitute formula	Infant formula supplemented with *B. lactis* Bb-12 (1 × 10^9^ CFU/g)	N/A	*Ad libitum* for 2 months	No data	Statistically significant reduction on SCORAD score in *B. lactis* Bb12 group	[85]
RDBPCT	50	7–24 months	Diagnosed with atopic dermatitis	*B. lactis* Bi-07 (1 × 10^10^ CFU)	N/A	Once daily for 8 weeks	Yes	Probiotic administration did not alter the composition of the microbiota, but an increase in *B. lactis* correlated with decreased SCORAD index, but could not be attributed to probiotic consumption	[86]
RDBPCT	208	3–6 months	Physician diagnosed ezcema	*B. lactis* CNCM I-3446 (1 × 10^10^ CFU)	Before supplementation 1% hydrocortisone ointment 2×/day, emollients/moisturisers 2–49/day, bath emollient	Once daily for 3 months	Yes	No benefit from supplementation with either bacteria compared with placebo	[87]
RDBPCT	75	Infants <7 months	Positive for atopic dermatitis	Whey formula containing *B. breve* M-16V (1.3 × 10^9^ CFU/100 ml) + 90% scGOS + 10% lcFOS, 0.8 g/100 ml	Topical steroids	On demand for 12 weeks	No data	Reduced asthma like symptoms and no. of subjects requiring asthma medication 1 year following Bif treatment compared with placebo	[88]
RDBPCT	77	18–75 years	Patients who satisfied Rome II criteria for IBS diagnosis	*B. infantis* 35 624 (1 × 10^10^ CFU)	N/A	Once daily for 8 weeks	Yes	Reduction in symptoms for Bif group. Normalised IL-10/IL-12 ratio when treated with Bif	[89]
RDBPCT	362		Women with bowel habit subtype	*B. infantis* 35 624 (1 × 10^6^ or 1 × 10^8^ CFU)	N/A	Once daily for 4 weeks		Reduction in symptom in 10^8^ CFU/ml Bif group compared with the placebo group	[90]
RDBPCT	122	18–68	Mild-to-moderate IBS (Rome III criteria)	*B. bifidum* MIMBb7 (1 × 10^9^)	N/A	Once daily for 4 weeks	No	Reduction in symptoms in the Bif treatment group	[91]

Abbreviations: RDBPCT, randomised; double-blind; placebo-controlled trial; RCT, randomised clinical trial; UC, ulcerative colitis; CD, Crohn's disease; Bif, *Bifidobacterium* supplemented; CAIA, clinical activity index assessment; GOS, galactooligosaccharide; scGOS, short-chain galactooligosaccharides; lcFOS, long-chain fructooligosaccharides; 5-ASA, 5-aminosalicylic acid; PPI, protein pump inhibitor; CRP, C-reactive protein.

### Inflammatory bowel diseases

IBD encompasses both Crohn's disease (CD) and ulcerative colitis (UC). Both diseases are characterised by chronic intestinal inflammation; UC inflammation is continuous from the rectum to the proximal colon, CD inflammation is patchy and discontinuous, and frequently occurs in the distal ileum or colon. The incidence of both these diseases is increasing in Western Europe and North America, and represents a significant burden on health services [92,93]. The aetiology of IBD is multifactorial, but it is widely accepted that the microbiota plays a key role in disease pathology. Patients with IBD have decreased microbial diversity, and many studies have shown a decrease in *Bifidobacterium* levels in both CD and UC patients during active disease [60,94–96] For a recent review on the topic, see Buttó & Haller [97].

Owing to the anti-inflammatory properties exhibited by many strains of *Bifidobacterium,* in conjunction with reduced levels of bifidobacteria in IBD, there have been several studies testing this bacteria as a treatment for IBD; one published trial for CD and six for UC (Table 2). However, a limited number of species (*B. longum* subsp. *longum*, *B. breve*, and *B. longum* subsp*. infantis*) have been used in these trials. Additionally, the treatment duration, number of patients, and disease makers studied in each trial vary greatly, and thus, comparison between trials is difficult. Despite these differences, the limited number of clinical trials shows some promise for using *Bifidobacterium* in the treatment of IBD. A pilot study in UC patients, which used a prebiotic (a fructo-oligosaccharide/inulin mix; Synergy 1) in conjunction with *B. longum* subsp. *longum* strain isolated from a healthy rectum, showed promising results despite low numbers of patients in the trial [73]. After 28-day treatment, patients in the treatment group had reduced *TNF* transcripts, a key cytokine in UC, and reduced clinical symptoms. A follow-up trial on patients with active CD, using the same probiotic/prebiotic mix, showed a reduction in CD activity index and histology score in patients receiving the synbiotic compared with the controls [82]. However, due to the short duration of these studies, it is not clear if this strain is effective in the induction or maintenance of remission, and whether a longer-term study would prove continued efficacy. In another short-term study, *B. longum* subsp*. infantis* 35 624 administered to UC patients for 6 weeks resulted in a significant reduction in C-reactive protein and a non-statistically significant reduction in IL-6 when compared with the baseline [70]. While this study indicated a decrease in inflammatory markers, no clinical outcomes were measured and therefore it is not possible to conclude that this strain is effective in the treatment of UC. More recently, a trial where patients with mild-to-moderate UC (UCDAI 3–9) were supplemented with *B. longum* subsp. *longum* 536 resulted in a significant decrease in disease activity following 8-week supplementation, whereas a significant decrease was not seen in the placebo group [84]. Taken together, these trials suggest that bifidobacteria may be a promising therapy for the treatment of IBD; however, the limitations of the studies must be considered. Many trails did not test whether the strain administered had colonised patients making it difficult to directly attribute an effect to the probiotic, or indeed, if the strain modulated the wider microbiota, as no microbiota profiling (i.e. 16S rRNA or shotgun metagenomics) was performed. In all trials, *Bifidobacterium* supplementation was additional to standard treatment therapies (e.g. immunosuppressants/aminosalicylates or steroids); therefore, the efficacy of bifidobacterial treatment alone is unclear. Furthermore, all trials reviewed had a low number of participants (<100) over a short duration, and larger clinical trials are needed to clarify the efficacy of bifidobacteria in treating IBD. The differences between strains studied, intervention time, frequency and concentration of dose, and the addition of synbiotic and clinical outcomes measured mean that studies are difficult to compare. Finally, two Cochrane reviews, focused on clinical trials testing the use of probiotics in the induction or remission of UC or CD, highlighted a lack of well-designed trials in this area. Furthermore, the authors could not make any conclusion about the efficacy of probiotics in the treatment of UC or CD [98,99]. Thus, a more robust standardised approach to clinical trials with *Bifidobacterium* species (and other probiotics) would benefit future studies.

### Irritable bowel syndrome

Another GI disorder that has been the focus of treatment with species of *Bifidobacterium* is irritable bowel syndrome (IBS). The pathophysiology and cause of IBS is not fully understood; however; there is an immune component, as IBS patients have higher serum cytokine levels [100]. *B. longum* subsp. *infantis* 35 624 has been studied in two double-blind, placebo-controlled clinical trials [89,90]. In both studies, the bifidobacteria-supplemented group had reduction in symptoms, and in one trial, a reduction in cytokine production by peripheral blood mononuclear cells (PBMCs) was reported *in vitro* [89]. These data suggest that at least in some conditions, bifidobacteria could be useful in the management of IBS.

### Atopic eczema and asthma

The intestinal microbiota is important in early life immune development, and disturbances via antibiotics usage, formula feeding, or C-section are proposed to contribute to extra-intestinal disease, such as asthma and atopic eczema [101]. Studies have shown that infants who develop atopy have a lower *Bifidobacterium* to *Clostridium difficile* ratio [102]. Several trials have tested the use of probiotics as an intervention for infants with eczema and asthma. In an intervention study, Van Der Aa et al. [88] found that supplementation of *B. breve* M-16V, plus a prebiotic, to infants less than 7 months old, who were positive for atopic eczema, resulted in less children on asthma medication 1-year post-treatment. The three studies of eczema carried out in infants under 24 months, who had developed atopic eczema, and had a variety of study designs, used the SCORAD (Scoring atopic dermatitis), allowing for some comparison between studies [85–87]. An early study focused on 3–6-month-old infants who had developed eczema during breast-feeding and had never been exposed to infant formula [85]. Children were provided with *Bifidobacterium animalis* subsp. *lactis* Bb-12 supplemented exclusively with hydrolysed whey formula for 2 months, resulting in a reduction in SCORAD from 16 to 0 vs. 13.4 in the supplement group. Another study supplementing *B. animalis* subsp. *lactis* Bi-07 to infants diagnosed with eczema resulted in a correlation between an increase in *Bifidobacterium* spp. in the infant microbiota and a decreased SCORAD index, but this could not be directly attributed to probiotic consumption [86]. While these two studies suggest that supplementation with bifidobacteria could help reduce the symptoms of atopic eczema, another larger, longer-term clinical trial showed no benefit of supplementation with *B. animalis* subsp. *lactis* CNCM I-3446, highlighting that not all clinical interventions with bifidobacteria are successful [87].

### Necrotising enterocolitis

NEC primarily occurs in premature, and low-birth-weight infants, and can result in death. These infants have an underdeveloped intestinal immune system and are given broad-spectrum antibiotics prophylactically to prevent infection. Colonisation with opportunistic pathogens may contribute to the pathogenesis of NEC, which is characterised by an exacerbated inflammatory cascade [103]. A recent study, where preterm infants were supplemented with *B. breve* M-16V, showed a significant reduction in NEC ≥ Stage II, highlighting a role for *Bifidobacterium* in this disease [104]. The mechanism of a bifidobacteria-protective effect in NEC is not clear, but one study in a rat NEC model showed that *B. bifidum* OLB6378 modulated mucosal immunity by reducing *Il6* and *Tnfa* expression, and improving TJ protein distribution in the ileum [107]. However, bifidobacteria are also known to inhibit pathogen colonisation [4] and thus may directly modulate the microbiota and inhibit NEC; however, more studies are required to clarify this. A recent large-scale study supplementing preterm infants with *B. breve* BBG-001 suggested that supplementation does not prevent NEC or late-onset sepsis in the study group [106]; however, the outcomes of this study remain controversial [107,108].

The above clinical trials have identified some positive roles for the treatment of immune-driven diseases with *Bifidobacterium* therapy; however, other studies have shown no benefit. Furthermore, there is a current lack of understanding, with respect to the underlying immune-modulatory factors involved in improving clinical outcomes. Currently, there is also a lack of bifidobacterial supplementation studies aimed at positively modulating other immune-linked conditions, such as arthritis and psoriasis. Further identifying the mechanisms by which bifidobacteria modulate the immune system in humans, building on in-depth mechanistic animal studies, will allow for better screening of new potential therapeutic strains. In IBD, with the highest number of trials, there is scope for better standardisation of secondary outcomes to allow for better comparison between independent studies (Box 2).

Box 2. **Recommendations for future *Bifidobacterium* intervention trials in human patients**

**Clinical study design recommendations**

• Profile colonisation ability of strain(s)

• Stratify responders vs. non-responders and cross-talk capabilities

• Determine the impact of supplementation on wider microbiota (e.g. 16S or shotgun)

• Define clear primary standardised clinical readouts

• Define clear immune markers associated with disease as secondary readouts, using markers from preclinical models

• Define clear microbiota and immune baselines for patients before intervention

• Longitudinal sampling throughout intervention

• Define cohort to be tested based on preclinical model data (e.g. paediatric vs. adult)

## Conclusion and future perspectives

The studies, to date, have shown that *Bifidobacterium* are resident within the GI tract across our lifespan, and are associated with immune well-being. Notably, reductions in bifidobacterial populations are associated with various immune-linked conditions, and studies using *in vivo* models and clinical trials indicate strategies that use *Bifidobacterium* may beneficially modulate immune responses to improve clinical symptoms. However, we are still somewhat removed from understanding how different strains of bifidobacteria specifically modulate immune responses (Box 1), and how we link this to comprehensive and well-planned clinical trials (Box 2). These studies are critical if we are to perform more personalised interventions in patients with immune-linked diseases, with the aim of improving clinical outcomes and providing cost-effective and potentially non-toxic therapies.

## Summary

- *Bifidobacterium* spp. are present in the human gastrointestinal tract from birth and throughout the life course, and their presence is associated with health.
- Reduction in bifidobacterial abundance occurs in multiple inflammatory diseases.
- Bifidobacteria can modulate T-cell responses to reduce inflammation.
- Bifidobacteria may modulate T cells indirectly through dendritic cells to reduce inflammation.
- The overall mechanisms of bifidobacterial-associated immune modulation are currently incompletely understood.
- Bifidobacteria supplementation to treat inflammatory diseases shows promise, but more studies are required.

## Abbreviations

CAIA: clinical activity index assessment
CD: Crohn's disease
C-section: caesarean section
CRP: C-reactive protein
DCs: dendritic cells
DSS: dextran sodium sulfate
EPS: exopolysaccharide
GH: glycosyl hydrolases
GI: gastrointestinal
GOS: galactooligosaccharides
IBD: inflammatory bowel disease
IECs: intestinal epithelial cells
ITS: internal transcribed spacer
LP: lamina propria
MLN: mesenteric lymph node
NEC: necrotising enterocolitis
NK: natural killer
PBMCs: peripheral blood mononuclear cells
SCORAD: scoring atopic dermatitis
Th1: T helper1
TJ: tight junction
TNBS: trinitrobenzene sulfonic acid
Tregs: T regulatory cells
UC: ulcerative colitis
ZO-1: zonula occludens-1

## References

[1] Power S.E., O'Toole P.W., Stanton C., Ross R.P. and Fitzgerald G.F. (2014) Intestinal microbiota, diet and health. Br. J. Nutr. 111, 387–402 10.1017/S0007114513002560 23931069

[2] Donaldson G.P., Lee S.M. and Mazmanian S.K. (2015) Gut biogeography of the bacterial microbiota. Nat. Rev. Microbiol. 14, 20–32 10.1038/nrmicro3552 26499895 PMC4837114

[3] Hill C., Guarner F., Reid G., Gibson G.R., Merenstein D.J., Pot B. et al. (2014) Expert consensus document: the International Scientific Association for Probiotics and Prebiotics consensus statement on the scope and appropriate use of the term probiotic. Nat. Rev. Gastroenterol. Hepatol. 11, 506–514 10.1038/nrgastro.2014.66 24912386

[4] Fukuda S., Toh H., Taylor T.D., Ohno H. and Hattori M. (2012) Acetate-producing bifidobacteria protect the host from enteropathogenic infection via carbohydrate transporters. Gut Microbes 3, 449–454 10.4161/gmic.21214 22825494

[5] Fanning S., Hall L.J., Cronin M., Zomer A., MacSharry J., Goulding D. et al. (2012) Bifidobacterial surface-exopolysaccharide facilitates commensal-host interaction through immune modulation and pathogen protection. Proc. Natl Acad. Sci. U.S.A. 109, 2108–2113 10.1073/pnas.1115621109 22308390 PMC3277520

[6] Asahara T., Shimizu K., Nomoto K., Hamabata T., Ozawa A. and Takeda Y. (2004) Probiotic bifidobacteria protect mice from lethal infection with shiga toxin-producing *Escherichia coli* O157:H7. Society 72, 2240–2247 PMID:15039348 10.1128/IAI.72.4.2240-2247.2004 PMC375161

[7] Cani P.D. and Delzenne N.M. (2009) Interplay between obesity and associated metabolic disorders: new insights into the gut microbiota. Curr. Opin. Pharmacol. 9, 737–743 10.1016/j.coph.2009.06.016 19628432

[8] Ling X., Linglong P., Weixia D. and Hong W. (2016) Protective effects of bifidobacterium on intestinal barrier function in LPS-induced enterocyte barrier injury of Caco-2 monolayers and in a rat NEC model. PLoS ONE 11, e0161635 10.1371/journal.pone.0161635 27551722 PMC4995054

[9] Corby-Harris V., Maes P. and Anderson K.E. (2014) The bacterial communities associated with honey bee (*Apis mellifera*) foragers. PLoS ONE 9, e95056 10.1371/journal.pone.0095056 24740297 PMC3989306

[10] Sharifpour M.F., Mardani K. and Ownagh A. (2016) Molecular identification and phylogenetic analysis of *Lactobacillus* and *Bifidobacterium* spp. isolated from gut of honeybees (*Apis mellifera*) from West Azerbaijan, Iran. Vet. Res. Forum. Int. Q. J. 7, 287–294 PMC:525135 PMC5251350 28144419

[11] Laureys D., Cnockaert M., De Vuyst L. and Vandamme P. (2016) *Bifidobacterium aquikefiri* sp. nov., isolated from water kefir. Int. J. Syst. Evol. Microbiol. 66, 1281–1286 10.1099/ijsem.0.000877 26739269

[12] Ventura M., O'Connell-Motherway M., Leahy S., Moreno-Munoz J.A., Fitzgerald G.F. and van Sinderen D. (2007) From bacterial genome to functionality; case bifidobacteria. Int. J. Food Microbiol. 120, 2–12 10.1016/j.ijfoodmicro.2007.06.011 17629975

[13] Klijn A., Mercenier A. and Arigoni F. (2005) Lessons from the genomes of bifidobacteria. FEMS Microbiol. Rev. 29, 491–509 10.1016/j.fmrre.2005.04.010 15939502

[14] Lugli G.A., Milani C., Turroni F., Duranti S., Mancabelli L., Mangifesta M. et al. (2017) Comparative genomic and phylogenomic analyses of the *Bifidobacteriaceae* family. BMC Genomics 18, 568 10.1186/s12864-017-3955-4 28764658 PMC5540593

[15] Lugli G.A., Milani C., Turroni F., Duranti S., Ferrario C., Viappiani A. et al. (2014) Investigation of the evolutionary development of the genus bifidobacterium by comparative genomics. Appl. Environ. Microbiol. 80, 6383–6394 10.1128/AEM.02004-14 25107967 PMC4178631

[16] Milani C., Turroni F., Duranti S., Lugli G.A., Mancabelli L., Ferrario C. et al. (2016) Genomics of the genus Bifidobacterium reveals species-specific adaptation to the glycan-rich gut environment. Appl. Environ. Microbiol. 82, 980–991 10.1128/AEM.03500-15 26590291 PMC4751850

[17] Arboleya S., Watkins C., Stanton C. and Ross R.P. (2016) Gut bifidobacteria populations in human health and aging. Front. Microbiol. 7, 1204 10.3389/fmicb.2016.01204 27594848 PMC4990546

[18] Nuriel-Ohayon M., Neuman H. and Koren O. (2016) Microbial changes during pregnancy, birth, and infancy. Front. Microbiol. 7, 1031 10.3389/fmicb.2016.01031 27471494 PMC4943946

[19] Makino H., Martin R., Ishikawa E., Gawad A., Kubota H., Sakai T. et al. (2015) Multilocus sequence typing of bifidobacterial strains from infant's faeces and human milk: are bifidobacteria being sustainably shared during breastfeeding? Benef. Microbes 6, 563–572 10.3920/BM2014.0082 25691099

[20] Collado M.C., Rautava S., Aakko J., Isolauri E. and Salminen S. (2016) Human gut colonisation may be initiated *in utero* by distinct microbial communities in the placenta and amniotic fluid. Sci. Rep. 6, 23129 10.1038/srep23129 27001291 PMC4802384

[21] Rautava S., Collado M.C., Salminen S. and Isolauri E. (2012) Probiotics modulate host-microbe interaction in the placenta and fetal gut: a randomized, double-blind, placebo-controlled trial. Neonatology 102, 178–184 10.1159/000339182 22776980

[22] Duranti S., Lugli G.A., Mancabelli L., Armanini F., Turroni F., James K. et al. (2017) Maternal inheritance of bifidobacterial communities and bifidophages in infants through vertical transmission. Microbiome 5, 66 10.1186/s40168-017-0282-6 28651630 PMC5485682

[23] Milani C., Mancabelli L., Lugli G.A., Duranti S., Turroni F., Ferrario C. et al. (2015) Exploring vertical transmission of bifidobacteria from mother to child. Appl. Environ. Microbiol. 81, 7078–7087 10.1128/AEM.02037-15 26231653 PMC4579462

[24] Dominguez-Bello M.G., Costello E.K., Contreras M., Magris M., Hidalgo G., Fierer N. et al. (2010) Delivery mode shapes the acquisition and structure of the initial microbiota across multiple body habitats in newborns. Proc. Natl Acad. Sci. U.S.A. 107, 11971–11975 10.1073/pnas.1002601107 20566857 PMC2900693

[25] Chen J., Cai W. and Feng Y. (2007) Development of intestinal bifidobacteria and lactobacilli in breast-fed neonates. Clin. Nutr. 26, 559–566 10.1016/j.clnu.2007.03.003 17507117

[26] Soto A., Martín V., Jiménez E., Mader I., Rodríguez J.M. and Fernández L. (2014) Lactobacilli and bifidobacteria in human breast milk: influence of antibiotherapy and other host and clinical factors. J. Pediatr. Gastroenterol. Nutr. 59, 78–88 10.1097/MPG.0000000000000347 24590211 PMC4086764

[27] Lewis Z.T. and Mills D.A. (2017) Differential establishment of bifidobacteria in the breastfed infant gut. Nestle Nutr. Inst. Workshop Ser. 88, 149–159 10.1159/000455399 28346936 PMC5535791

[28] Bode L. (2012) Human milk oligosaccharides: every baby needs a sugar mama. Glycobiology 22, 1147–1162 10.1093/glycob/cws074 22513036 PMC3406618

[29] Garrido D., Dallas D.C. and Mills D.A. (2013) Consumption of human milk glycoconjugates by infant-associated bifidobacteria: mechanisms and implications. Microbiology 159(Pt 4), 649–664 10.1099/mic.0.064113-0 23460033 PMC4083661

[30] Ly N.P., Litonjua A., Gold D.R. and Celedón J.C. (2011) Gut microbiota, probiotics, and vitamin D: interrelated exposures influencing allergy, asthma, and obesity? J. Allergy Clin. Immunol. 127, 1087–10 94; quiz 1095–1096 10.1016/j.jaci.2011.02.015 21419479 PMC3085575

[31] Turroni F., Peano C., Pass D.A., Foroni E., Severgnini M., Claesson M.J. et al. (2012) Diversity of bifidobacteria within the infant gut microbiota. PLoS ONE 7, e36957 10.1371/journal.pone.0036957 22606315 PMC3350489

[32] Voreades N., Kozil A. and Weir T.L. (2014) Diet and the development of the human intestinal microbiome. Front. Microbiol. 5, 494 10.3389/fmicb.2014.00494 25295033 PMC4170138

[33] Yatsunenko T., Rey F.E., Manary M.J., Trehan I., Dominguez-Bello M.G., Contreras M. et al. (2012) Human gut microbiome viewed across age and geography. Nature 486, 222–227 10.1038/nature11053 22699611 PMC3376388

[34] Koenig J.E., Spor A., Scalfone N., Fricker A.D., Stombaugh J., Knight R. et al. (2011) Succession of microbial consortia in the developing infant gut microbiome. Proc. Natl Acad. Sci. U.S.A. 108(suppl 1), 4578–4585 10.1073/pnas.1000081107 20668239 PMC3063592

[35] Biagi E., Candela M., Fairweather-Tait S., Franceschi C. and Brigidi P. (2012) Ageing of the human metaorganism: the microbial counterpart. AGE 34, 247–267 10.1007/s11357-011-9217-5 21347607 PMC3260362

[36] Wang F., Huang G., Cai D., Li D., Liang X., Yu T. et al. (2015) Qualitative and semiquantitative analysis of fecal bifidobacterium species in centenarians living in Bama, Guangxi, China. Curr. Microbiol. 71, 143–149 10.1007/s00284-015-0804-z 26003628

[37] Miller L.E., Lehtoranta L. and Lehtinen M.J. (2017) The effect of *Bifidobacterium animalis* ssp. lactis HN019 on cellular immune function in healthy elderly subjects: systematic review and meta-analysis. Nutrients 9, 191 10.3390/nu9030191 PMC5372854 28245559

[38] Ruiz L., Delgado S., Ruas-Madiedo P., Margolles A. and Sánchez B. (2016) Proteinaceous molecules mediating *Bifidobacterium*-host interactions. Front. Microbiol. 7, 1193 10.3389/fmicb.2016.01193 27536282 PMC4971063

[39] López P., González-Rodríguez I., Gueimonde M., Margolles A. and Suárez A. (2011) Immune response to *Bifidobacterium bifidum* strains support Treg/Th17 plasticity. PLoS ONE 6, e24776 10.1371/journal.pone.0024776 21966367 PMC3178565

[40] Hughes K.R., Harnisch L.C., Alcon-Giner C., Mitra S., Wright C.J., Ketskemety J. et al. (2017) *Bifidobacterium breve* reduces apoptotic epithelial cell shedding in an exopolysaccharide and MyD88-dependent manner. Open Biol. 7, 160155 10.1098/rsob.160155 28123052 PMC5303268

[41] Meng D., Zhu W., Ganguli K., Shi H.N. and Walker W.A. (2016) Anti-inflammatory effects of *Bifidobacterium longum* subsp *infantis* secretions on fetal human enterocytes are mediated by TLR-4 receptors. Am. J. Physiol. Gastrointest. Liver Physiol. 311, G744–G753 10.1152/ajpgi.00090.2016 27562058 PMC5142200

[42] Turroni F., Serafini F., Foroni E., Duranti S., O'Connell Motherway M., Taverniti V. et al. (2013) Role of sortase-dependent pili of *Bifidobacterium bifidum* PRL2010 in modulating bacterium-host interactions. Proc. Natl Acad. Sci. U.S.A. 110, 11151–11156 10.1073/pnas.1303897110 23776216 PMC3703987

[43] Schiavi E., Gleinser M., Molloy E., Groeger D., Frei R., Ferstl R. et al. (2016) The surface-associated exopolysaccharide of *Bifidobacterium longum* 35624 plays an essential role in dampening host proinflammatory responses and repressing local T_H_17 responses. Appl. Environ. Microbiol. 82, 7185–7196 10.1128/AEM.02238-16 27736791 PMC5118929

[44] Jameson S.C. (2002) Maintaining the norm: T-cell homeostasis. Nat. Rev. Immunol. 2, 547 10.1038/nri853 12154374

[45] Hsu P. and Nanan R. (2014) Foetal immune programming: hormones, cytokines, microbes and regulatory T cells. J. Reprod. Immunol. 104–105, 2–7 10.1016/j.jri.2014.02.005 24702950

[46] Damsker J.M., Hansen A.M. and Caspi R.R. (2010) Th1 and Th17 cells: adversaries and collaborators. Ann. N. Y. Acad. Sci. 1183, 211–221 10.1111/j.1749-6632.2009.05133.x 20146717 PMC2914500

[47] Licona-Limón P., Kim L.K., Palm N.W. and Flavell R.A. (2013) TH2, allergy and group 2 innate lymphoid cells. Nat. Immunol. 14, 536–542 10.1038/ni.2617 23685824

[48] Wang Y., Liu X.P., Zhao Z.B., Chen J.H. and Yu C.G. (2011) Expression of CD4^+^ forkhead box P3 (FOXP3)^+^ regulatory T cells in inflammatory bowel disease. J. Dig. Dis. 12, 286–294 10.1111/j.1751-2980.2011.00505.x 21791023

[49] Sagar S., Morgan M.E., Chen S., Vos A.P., Garssen J., van Bergenhenegouwen J. et al. (2014) *Bifidobacterium breve* and *Lactobacillus rhamnosus* treatment is as effective as budesonide at reducing inflammation in a murine model for chronic asthma. Respir. Res. 15, 46 10.1186/1465-9921-15-46 24735374 PMC4029990

[50] van Esch B.C.A.M., Abbring S., Diks M.A.P., Dingjan G.M., Harthoorn L.F., Vos A.P. et al. (2016) Post-sensitization administration of non-digestible oligosaccharides and *Bifidobacterium breve* M-16V reduces allergic symptoms in mice. Immunity Inflamm. Dis. 4, 155–165 10.1002/iid3.101 PMC4879462 27933160

[51] Zheng B., van Bergenhenegouwen J., Overbeek S., van de Kant H.J.G., Garssen J., Folkerts G. et al. (2014) *Bifidobacterium breve* attenuates murine dextran sodium sulfate-induced colitis and increases regulatory T cell responses. PLoS ONE 9, e95441 10.1371/journal.pone.0095441 24787575 PMC4008378

[52] Zuo L., Yuan K.-T., Yu L., Meng Q.-H., Chung P.C.-K. and Yang D.-H. (2014) *Bifidobacterium infantis* attenuates colitis by regulating T cell subset responses. World J. Gastroenterol. 20, 18316–18329 10.3748/wjg.v20.i48.18316 25561798 PMC4277968

[53] Zhang D., Wei C., Yao J., Cai X. and Wang L. (2015) Interleukin-10 gene-carrying bifidobacteria ameliorate murine ulcerative colitis by regulating regulatory T cell/T helper 17 cell pathway. Exp. Biol. Med. 240, 1622–1629 10.1177/1535370215584901 PMC4935350 25956685

[54] Jeon S.G., Kayama H., Ueda Y., Takahashi T., Asahara T., Tsuji H. et al. (2012) Probiotic *Bifidobacterium breve* induces IL-10-producing Tr1 cells in the colon. PLoS Pathog. 8, e1002714 10.1371/journal.ppat.1002714 22693446 PMC3364948

[55] Konieczna P., Ferstl R., Ziegler M., Frei R., Nehrbass D., Lauener R.P. et al. (2013) Immunomodulation by *Bifidobacterium infantis* 35624 in the murine lamina propria requires retinoic acid-dependent and independent mechanisms. PLoS ONE 8, e62617 10.1371/journal.pone.0062617 23704880 PMC3660574

[56] Yang J., Zhang H., Jiang L., Guo H., Luo X. and Ren F. (2015) *Bifidobacterium longum* BBMN68-specific modulated dendritic cells alleviate allergic responses to bovine β -lactoglobulin in mice. J. Appl. Microbiol. 119, 1127–1137 10.1111/jam.12923 26248977

[57] Hsieh C.-Y., Osaka T., Moriyama E., Date Y., Kikuchi J. and Tsuneda S. (2015) Strengthening of the intestinal epithelial tight junction by *Bifidobacterium bifidum*. Physiol. Rep. 3, e12327 10.14814/phy2.12327 25780093 PMC4393161

[58] Ewaschuk J.B., Diaz H., Meddings L., Diederichs B., Dmytrash A., Backer J. et al. (2008) Secreted bioactive factors from *Bifidobacterium infantis* enhance epithelial cell barrier function. Am. J. Physiol. Gastrointest. Liver Physiol. 295, G1025–G1034 10.1152/ajpgi.90227.2008 18787064

[59] Lopez P., Gonzalez-Rodriguez I., Sanchez B., Ruas-Madiedo P., Suarez A., Margolles A. et al. (2012) Interaction of *Bifidobacterium bifidum* LMG13195 with HT29 cells influences regulatory-T-cell-associated chemokine receptor expression. Appl. Environ. Microbiol. 78, 2850–2857 10.1128/AEM.07581-11 22344636 PMC3318848

[60] Macfarlane S., Furrie E., Cummings J.H. and Macfarlane G.T. (2004) Chemotaxonomic analysis of bacterial populations colonizing the rectal mucosa in patients with ulcerative colitis. Clin. Infect. Dis. 38, 1690–1699 10.1086/420823 15227614

[61] Williams J.M., Duckworth C.A., Burkitt M.D., Watson A.J.M., Campbell B.J. and Pritchard D.M. (2015) Epithelial cell shedding and barrier function: a matter of life and death at the small intestinal villus tip. Vet. Pathol. 52, 445–455 10.1177/0300985814559404 25428410 PMC4441880

[62] Kiesslich R., Duckworth C.A., Moussata D., Gloeckner A., Lim L.G., Goetz M. et al. (2012) Local barrier dysfunction identified by confocal laser endomicroscopy predicts relapse in inflammatory bowel disease. Gut 61, 1146–1153 10.1136/gutjnl-2011-300695 22115910 PMC3388727

[63] Srutkova D., Schwarzer M., Hudcovic T., Zakostelska Z., Drab V., Spanova A. et al. (2015) *Bifidobacterium longum* CCM 7952 promotes epithelial barrier function and prevents acute DSS-induced colitis in strictly strain-specific manner. PLoS ONE 10, e0134050 10.1371/journal.pone.0134050 26218526 PMC4517903

[64] Grimm V., Radulovic K. and Riedel C.U. (2015) Colonization of C57BL/6 mice by a potential probiotic *Bifidobacterium bifidum* strain under germ-free and specific pathogen-free conditions and during experimental colitis. PLoS ONE 10, e0139935 10.1371/journal.pone.0139935 26439388 PMC4595203

[65] Kawahara T., Takahashi T., Oishi K., Tanaka H., Masuda M., Takahashi S. et al. (2015) Consecutive oral administration of *Bifidobacterium longum* MM-2 improves the defense system against influenza virus infection by enhancing natural killer cell activity in a murine model. Microbiol. Immunol. 59, 1–12 10.1111/1348-0421.12210 25400245

[66] Moya-Pérez A., Neef A. and Sanz Y. (2015) *Bifidobacterium pseudocatenulatum* CECT 7765 reduces obesity-associated inflammation by restoring the lymphocyte-macrophage balance and gut microbiota structure in high-fat diet-fed mice. PLoS ONE 10, e0126976 10.1371/journal.pone.0126976 26161548 PMC4498624

[67] Iwabuchi N., Takahashi N., Xiao J.-z., Miyaji K. and Iwatsuki K. (2007) *In vitro* Th1 cytokine-independent Th2 suppressive effects of bifidobacteria. Microbiol. Immunol. 51, 649–660 10.1111/j.1348-0421.2007.tb03953.x 17641467

[68] Medina M., De Palma G., Ribes-Koninckx C., Calabuig M. and Sanz Y. (2008) Bifidobacterium strains suppress in vitro the pro-inflammatory milieu triggered by the large intestinal microbiota of coeliac patients. J. Inflamm. 5, 19 10.1186/1476-9255-5-19 PMC2640389 18980693

[69] Lim S.-M. and Kim D.-H. (2017) *Bifidobacterium adolescentis* IM38 ameliorates high-fat dietiinduced colitis in mice by inhibiting NF-κB activation and lipopolysaccharide production by gut microbiota. Nutr. Res. 41, 86–96 10.1016/j.nutres.2017.04.003 28479226

[70] Groeger D., O'Mahony L., Murphy E.F., Bourke J.F., Dinan T.G., Kiely B. et al. (2013) *Bifidobacterium infantis* 35624 modulates host inflammatory processes beyond the gut. Gut Microbes 4, 325–339 10.4161/gmic.25487 23842110 PMC3744517

[71] Preising J., Philippe D., Gleinser M., Wei H., Blum S., Eikmanns B.J. et al. (2010) Selection of bifidobacteria based on adhesion and anti-inflammatory capacity in vitro for amelioration of murine colitis. Appl. Environ. Microbiol. 76, 3048–3051 10.1128/AEM.03127-09 20228095 PMC2863435

[72] McCarthy J., O'Mahony L., O'Callaghan L., Sheil B., Vaughan E.E., Fitzsimons N. et al. (2003) Double blind, placebo controlled trial of two probiotic strains in interleukin 10 knockout mice and mechanistic link with cytokine balance. Gut 52, 975–980 10.1136/gut.52.7.975 12801954 PMC1773705

[73] Furrie E., Macfarlane S., Kennedy A., Cummings J.H., Walsh S.V., O'neil D.A. et al. (2005) Synbiotic therapy (*Bifidobacterium longum*/Synergy 1) initiates resolution of inflammation in patients with active ulcerative colitis: a randomised controlled pilot trial. Gut 54, 242–249 10.1136/gut.2004.044834 15647189 PMC1774839

[74] O'Mahony L., O'Callaghan L., McCarthy J., Shilling D., Scully P., Sibartie S. et al. (2006) Differential cytokine response from dendritic cells to commensal and pathogenic bacteria in different lymphoid compartments in humans. Am. J. Physiol. Gastrointest. Liver Physiol. 290, G839–G845 10.1152/ajpgi.00112.2005 16293657

[75] Kim N., Kunisawa J., Kweon M.-N., Eog Ji G. and Kiyono H. (2007) Oral feeding of *Bifidobacterium bifidum* (BGN4) prevents CD4+ CD45RBhigh T cell-mediated inflammatory bowel disease by inhibition of disordered T cell activation. Clin. Immunol. 123, 30–39 10.1016/j.clim.2006.11.005 17218154

[76] Imaoka A., Shima T., Kato K., Mizuno S., Uehara T., Matsumoto S. et al. (2008) Anti-inflammatory activity of probiotic *Bifidobacterium*: enhancement of IL-10 production in peripheral blood mononuclear cells from ulcerative colitis patients and inhibition of IL-8 secretion in HT-29 cells. World J. Gastroenterol. 14, 2511–2516 10.3748/wjg.14.2511 18442197 PMC2708361

[77] Philippe D., Heupel E., Blum-Sperisen S. and Riedel C.U. (2011) Treatment with *Bifidobacterium bifidum* 17 partially protects mice from Th1-driven inflammation in a chemically induced model of colitis. Int. J. Food Microbiol. 149, 45–49 10.1016/j.ijfoodmicro.2010.12.020 21257218

[78] Sheikhi A., Shakerian M., Giti H., Baghaeifar M., Jafarzadeh A., Ghaed V. et al. (2016) Probiotic yogurt culture *Bifidobacterium animalis* subsp. lactis BB-12 and *Lactobacillus acidophilus* LA-5 modulate the cytokine secretion by peripheral blood mononuclear cells from patients with ulcerative colitis. Drug Res. 66, 300–305 10.1055/s-0035-1569414 26909690

[79] Duranti S., Gaiani F., Mancabelli L., Milani C., Grandi A., Bolchi A. et al. (2016) Elucidating the gut microbiome of ulcerative colitis: bifidobacteria as novel microbial biomarkers. FEMS Microbiol. Ecol. 92, fiw191 10.1093/femsec/fiw191 27604252

[80] Kamada N., Sakamoto K., Seo S.-U., Zeng M.Y., Kim Y.-G., Cascalho M. et al. (2015) Humoral immunity in the gut selectively targets phenotypically virulent attaching-and-effacing bacteria for intraluminal elimination. Cell Host Microbe 17, 617–627 10.1016/j.chom.2015.04.001 25936799 PMC4433422

[81] Fujimori S., Gudis K., Mitsui K., Seo T., Yonezawa M., Tanaka S. et al. (2009) A randomized controlled trial on the efficacy of synbiotic versus probiotic or prebiotic treatment to improve the quality of life in patients with ulcerative colitis. Nutrition 25, 520–525 10.1016/j.nut.2008.11.017 19201576

[82] Steed H., MacFarlane G.T., Blackett K.L., Bahrami B., Reynolds N., Walsh S.V. et al. (2010) Clinical trial: the microbiological and immunological effects of synbiotic consumption — a randomized double-blind placebo-controlled study in active Crohn's disease. Aliment Pharmacol. Ther. 32, 872–883 10.1111/j.1365-2036.2010.04417.x 20735782

[83] Ishikawa H., Matsumoto S., Ohashi Y., Imaoka A., Setoyama H., Umesaki Y. et al. (2011) Beneficial effects of probiotic *Bifidobacterium* and galacto-oligosaccharide in patients with ulcerative colitis: a randomized controlled study. Digestion 84, 128–133 10.1159/000322977 21525768

[84] Tamaki H., Nakase H., Inoue S., Kawanami C., Itani T., Ohana M. et al. (2016) Efficacy of probiotic treatment with *Bifidobacterium longum 536* for induction of remission in active ulcerative colitis: a randomized, double-blinded, placebo-controlled multicenter trial. Dig. Endosc. 28, 67–74 10.1111/den.12553 26418574

[85] Isolauri E., Arvola T., Sütas Y., Moilanen E. and Salminen S. (2000) Probiotics in the management of atopic eczema. Clin. Exp. Allergy 30, 1604–1610 10.1046/j.1365-2222.2000.00943.x 11069570

[86] Larsen N., Vogensen F.K., Gøbel R., Michaelsen K.F., Al-Soud W.A., Sørensen S.J. et al. (2011) Predominant genera of fecal microbiota in children with atopic dermatitis are not altered by intake of probiotic bacteria *Lactobacillus acidophilus* NCFM and *Bifidobacterium animalis* subsp. lactis Bi-07. FEMS Microbiol. Ecol. 75, 482–496 10.1111/j.1574-6941.2010.01024.x 21204871

[87] Gore C., Custovic A., Tannock G.W., Munro K., Kerry G., Johnson K. et al. (2012) Treatment and secondary prevention effects of the probiotics *Lactobacillus paracasei* or *Bifidobacterium lactis* on early infant eczema: randomized controlled trial with follow-up until age 3 years. Clin. Exp. Allergy 42, 112–122 10.1111/j.1365-2222.2011.03885.x 22092692

[88] Van Der Aa L.B., Van Aalderen W.M.C., Heymans H.S.A., Henk Sillevis Smitt J., Nauta A.J., Knippels L.M.J. et al. (2011) Synbiotics prevent asthma-like symptoms in infants with atopic dermatitis. Allergy Eur. J. Allergy Clin. Immunol. 66, 170–177 10.1111/j.1398-9995.2010.02416.x 20560907

[89] O'Mahony L., McCarthy J., Kelly P., Hurley G., Luo F., Chen K. et al. (2005) Lactobacillus and bifidobacterium in irritable bowel syndrome: symptom responses and relationship to cytokine profiles. Gastroenterology 128, 541–551 10.1053/j.gastro.2004.11.050 15765388

[90] Whorwell P.J., Altringer L., Morel J., Bond Y., Charbonneau D., O'Mahony L. et al. (2006) Efficacy of an encapsulated probiotic *Bifidobacterium infantis* 35624 in women with irritable bowel syndrome. Am. J. Gastroenterol. 101, 1581–1590 10.1111/j.1572-0241.2006.00734.x 16863564

[91] Guglielmetti S., Mora D., Gschwender M. and Popp K. (2011) Randomised clinical trial: *Bifidobacterium bifidum* MIMBb75 significantly alleviates irritable bowel syndrome and improves quality of life — a double-blind, placebo-controlled study. Aliment. Pharmacol. Ther. 33, 1123–1132 10.1111/j.1365-2036.2011.04633.x 21418261

[92] Ghosh N. and Premchand P. (2015) A UK cost of care model for inflammatory bowel disease. Frontline Gastroenterol. 6, 169–174 10.1136/flgastro-2014-100514 28839807 PMC5369575

[93] Mehta F. (2016) Report: economic implications of inflammatory bowel disease and its management. Am. J. Manag. Care 22(3 Suppl), s51–s60 PMID:27269903

[94] Mylonaki M., Rayment N.B., Rampton D.S., Hudspith B.N. and Brostoff J. (2005) Molecular characterization of rectal mucosa-associated bacterial flora in inflammatory bowel disease. Inflamm. Bowel Dis. 11, 481–487 10.1097/01.MIB.0000159663.62651.4f 15867588

[95] Macfarlane S., Furrie E., Kennedy A., Cummings J.H. and Macfarlane G.T. (2005) Mucosal bacteria in ulcerative colitis. Br. J. Nutr. 93, S67 10.1079/BJN20041347 15877898

[96] Gueimonde M., Ouwehand A., Huhtinen H., Salminen E. and Salminen S. (2007) Qualitative and quantitative analyses of the bifidobacterial microbiota in the colonic mucosa of patients with colorectal cancer, diverticulitis and inflammatory bowel disease. World J. Gastroenterol. 13, 3985–3989 10.3748/wjg.v13.i29.3985 17663515 PMC4171173

[97] Buttó L.F. and Haller D. (2017) Intestinal microbiology and ecology in Crohn's disease and ulcerative colitis In Crohn's Disease and Ulcerative Colitis: From Epidemiology and Immunobiology to a Rational Diagnostic and Therapeutic Approach Cham (Baumgart D.C., ed.), pp. 67–74. Springer International Publishing

[98] Naidoo K., Gordon M., Fagbemi A.O., Thomas A.G. and Akobeng A.K. (2011) Probiotics for maintenance of remission in ulcerative colitis. Cochrane Database Syst. Rev. Issue 12, CD007443 10.1002/14651858.CD007443.pub2 22161412

[99] Butterworth A.D., Thomas A.G. and Akobeng A.K. (2008) Probiotics for induction of remission in Crohn's disease. Cochrane Database Syst. Rev. Issue 3, CD006634 10.1002/14651858.CD006634.pub2 18646162 PMC6544811

[100] Seyedmirzaee S., Hayatbakhsh M.M., Ahmadi B., Baniasadi N., Bagheri Rafsanjani A.M., Nikpoor A.R. et al. (2016) Serum immune biomarkers in irritable bowel syndrome. Clin. Res. Hepatol. Gastroenterol. 40, 631–637 10.1016/j.clinre.2015.12.013 26850360

[101] Fujimura K.E. and Lynch S.V. (2015) Microbiota in allergy and asthma and the emerging relationship with the gut microbiome. Cell Host Microbe 17, 592–602 10.1016/j.chom.2015.04.007 25974301 PMC4443817

[102] Kalliomäki M., Collado M.C., Salminen S. and Isolauri E. (2008) Early differences in fecal microbiota composition in children may. Am. J. Clin. Nutr. 87, 534–538 PMID:18326589 10.1093/ajcn/87.3.534

[103] Coggins S.A., Wynn J.L. and Weitkamp J.-H. (2015) Infectious causes of necrotizing enterocolitis. Clin. Perinatol. 42, 133–154 10.1016/j.clp.2014.10.012 25678001 PMC4328138

[104] Patole S.K., Rao S.C., Keil A.D., Nathan E.A., Doherty D.A. and Simmer K.N. (2016) Benefits of *Bifidobacterium breve* M-16V supplementation in preterm neonates — a retrospective cohort study. PLoS ONE 11, e0150775 10.1371/journal.pone.0150775 26953798 PMC4783036

[105] Khailova L., Dvorak K., Arganbright K.M., Halpern M.D., Kinouchi T., Yajima M. et al. (2009) *Bifidobacterium bifidum* improves intestinal integrity in a rat model of necrotizing enterocolitis. Am J Physiol Gastrointest. Liver Physiol. 297, G940–G949 10.1152/ajpgi.00141.2009 20501441 PMC2777452

[106] Costeloe K., Hardy P., Juszczak E., Wilks M. and Millar M.R. (2016) *Bifidobacterium breve* BBG-001 in very preterm infants: a randomised controlled phase 3 trial. Lancet 387, 649–660 10.1016/S0140-6736(15)01027-2 26628328

[107] McKinlay C.J.D., Rebello C. and Tarnow-Mordi W. (2016) Probiotics in very preterm infants: the PiPS trial. Lancet 388, 655 10.1016/S0140-6736(16)31270-3 27533429

[108] Deshpande G., Rao S., Athalye-Jape G., Conway P. and Patole S. (2016) Probiotics in very preterm infants: the PiPS trial. Lancet 388, 655 10.1016/S0140-6736(16)31271-5 27533430

